# AMPA Receptor Activation Causes Silencing of AMPA Receptor-Mediated Synaptic Transmission in the Developing Hippocampus

**DOI:** 10.1371/journal.pone.0034474

**Published:** 2012-04-02

**Authors:** Pontus Wasling, Joakim Strandberg, Eric Hanse

**Affiliations:** Department of Physiology, Institute of Neuroscience and Physiology, The Sahlgrenska Academy, University of Gothenburg, Gothenburg, Sweden; Institut National de la Santé et de la Recherche Médicale, France

## Abstract

Agonist-induced internalization of transmembrane receptors is a widespread biological phenomenon that also may serve as a mechanism for synaptic plasticity. Here we show that the agonist AMPA causes a depression of AMPA receptor (AMPAR) signaling at glutamate synapses in the CA1 region of the hippocampus in slices from developing, but not from mature, rats. This developmentally restricted agonist-induced synaptic depression is expressed as a total loss of AMPAR signaling, without affecting NMDA receptor (NMDAR) signaling, in a large proportion of the developing synapses, thus creating AMPAR silent synapses. The AMPA-induced AMPAR silencing is induced independently of activation of mGluRs and NMDARs, and it mimics and occludes stimulus-induced depression, suggesting that this latter form of synaptic plasticity is expressed as agonist-induced removal of AMPARs. Induction of long-term potentiation (LTP) rendered the developing synapses resistant to the AMPA-induced depression, indicating that LTP contributes to the maturation-related increased stability of these synapses. Our study shows that agonist binding to AMPARs is a sufficient triggering stimulus for the creation of AMPAR silent synapses at developing glutamate synapses.

## Introduction

Activity-dependent changes in synaptic strength, synaptic plasticity, is believed to be decisive both for formation of the basal synaptic organization during brain development, as well as for learning throughout life. At glutamate synapses, insertion or removal of synaptic alpha-amino-3-hydroxy-5-methyl-4-isoxazolepropionic receptors (AMPARs) is a major mechanism underlying synaptic plasticity in learning as well as in development [1], [2]. Activation of *N*-methyl-D-aspartate (NMDA) or metabotropic glutamate receptors (NMDAR or mGluRs) are important triggers for alterations in AMPAR trafficking to or from the postsynaptic plasma membrane [1], [2], but the relevance of activation of the AMPARs themselves has received less attention.

It is becoming increasingly clear that the molecular mechanisms that regulate both induction and expression of synaptic plasticity may differ substantially between developing and mature synapses [3]–[7]. Thus, there are numerous differences between nascent and mature synapses, including the type of membrane receptor that triggers induction of synaptic plasticity, the intracellular pathways involved, as well as mechanisms for AMPAR trafficking. One particular salient difference between developing and mature synaptic circuits is the continuous formation and elimination of synapses in the developing brain [8]–[11]). Although generation of synapses can occur independently of synaptic activity [12], stabilization and elimination are thought to be initiated by developmental forms of long-term potentiation (LTP) and long-term depression (LTD), respectively [13], [14]. One form of LTD, which is likely important in initiating activity-dependent synaptic pruning during the developmental period, is expressed as removal of synaptic AMPARs [15]. This LTD can also be induced by application of agonists to NMDARs and mGluRs [15]. Chemical induction of LTD is experimentally advantageous as it affects most synapses in the preparation, which considerably facilitates the examination of the molecular mechanisms underlying synaptic plasticity. Similar to chemical LTD, application of the agonist AMPA may also remove AMPARs from the synapse [16]–[18], but agonist-induced removal of AMPARs has yet no counterpart in synaptic plasticity. Rather, agonist-induced removal of AMPARs has been linked to the constitutive recycling of AMPARs [18]–[20].

One form of synaptic plasticity that might use the mechanisms of agonist-induced internalization of AMPARs is activity-induced silencing of AMPA receptor-mediated transmission, resulting in AMPA silent synapses during early brain development [21]–[25]. This activity-induced AMPAR silencing is confined to the developmental period at glutamate synapses onto principal neurons [21], [22], but it persists into adulthood at glutamate synapses onto interneurons [26]. In the developing rat hippocampus (first two postnatal weeks) synaptic activation is sufficient to induce AMPAR silencing in a large proportion of glutamate synapses, and this AMPAR silencing could be readily reversed, or unsilenced by conventional LTP induction protocols [21], [22], [27]. Even synaptic activation at very low frequencies (0.05–0.2 Hz), and notably in the absence of NMDAR or mGluR activation, is sufficient to induce AMPAR silencing [21], [26], [27]. These results suggest that AMPAR activation *per se* may be a sufficient inducing stimulus for this form of plasticity. In the present study, we tested this idea by activating AMPARs directly with the agonist AMPA and we found that AMPA itself induces removal of synaptic AMPAR signaling, leaving NMDAR signaling unaffected. Moreover, agonist-induced silencing occludes further activity-induced AMPAR silencing at developing synapses onto CA1 pyramidal neurons. At mature synapses, neither agonist-induced depression nor stimulus-induced depression was present.

## Materials and Methods

### Ethics statement

This study was specifically approved by the Gothenburg ethical committee for animal research (Approval ID: Dnr 201-2010) and the animals were kept and killed in accordance with their specific guidelines.

### Drugs

Drugs were purchased from Sigma-Aldrich (Stockholm, Sweden), except for D-AP5 (Ascent Scientific, Bristol, UK) and (RS)-AMPA hydrobromide (Tocris Cookson, Bristol, UK).

### Electrophysiology

Experiments were performed on hippocampal slices from Wistar rats of either sex 7–13 day-old and male rats 26–46 day-old. The rats were anaesthetized with isoflurane prior to decapitation. After removal, the brain was placed in an ice-cold solution containing (in mM): 140 cholineCl, 2.5 KCl, 0.5 CaCl_2_, 7 MgCl_2_, 25 NaHCO_3_, 1.25 NaH_2_PO_4_, 1.3 ascorbic acid, and 7 dextrose. Transverse hippocampal slices, 300–400 µm thick, were cut with a vibratome (HM 650 V, Microm, Walldorf, Germany) in the same ice-cold solution and they were subsequently stored in artificial cerebrospinal fluid (ACSF) containing (in mM) 124 NaCl, 3 KCl, 2 CaCl_2_, 4 MgCl_2_, 26 NaHCO_3_, 1.25 NaH_2_PO_4_, 0.5 ascorbic acid, 3 myo-inositol, 4 D, L-lactic acid, and 10 D-glucose. After 1–5 hours of storage, a single slice was placed in a submersion recording chamber where it was constantly perfused with ACSF at a constant flow (∼2 ml minute^−1^) at room temperature. The perfusion ACSF contained (in mM) 124 NaCl, 3 KCl, 4 CaCl_2_, 4 MgCl_2_, 26 NaHCO_3_, 1.25 NaH_2_PO_4_, and 10 D-glucose. Picrotoxin (100 µM) was always present in the perfusion ACSF to block GABA_A_ receptors. All solutions were continuously bubbled with 95% O_2_ and 5% CO_2_ (pH ∼7.4).

Electrical stimulation of Schaffer collateral afferents was applied at 0.2 Hz in the stratum radiatum. Stimulation impulses consisted of 200 µs constant current pulses generated by a stimulator (Model DS3, Digitimer Ltd, Letchworth Garden City, UK) and delivered through an insulated tungsten microelectrode (resistance ∼0.5 MΩ). Whole-cell patch-clamp recordings were performed on visually identified pyramidal cells, using infrared differential interference contrast video microscopy mounted on an Olympus BX51WI microscope. The pipette solution contained (in mM) 130 Cs-methanesulfonate, 2 NaCl, 20 HEPES, 0.6 EGTA, 5 QX-314, 4 Mg-ATP, and 0.4 GTP (pH ∼7.3 and osmolality 280–300 mOsm). Liquid junction potential was measured to be about 8 mV and was not corrected for. Patch pipette resistance was 3–6 MΩ. Field EPSP (fEPSP) recordings were made by means of a glass micropipette filled with 1 M NaCl (resistance ∼2 MΩ) in the stratum radiatum. EPSCs and fEPSPs were recorded at a sampling frequency of 10 kHz and filtered at 1 kHz, using an EPC-9 amplifier (HEKA Elektronik, Lambrecht, Germany) and binned in groups of three synaptic responses. For AMPA EPSC recordings, cells were held in voltage-clamp mode at −70 mV. Series resistance was monitored using a 5 ms 10 mV hyperpolarizing pulse and was not allowed to change more than 20% in whole-cell experiments. EPSCs and fEPSPs were analyzed off-line using custom made IGOR Pro (WaveMetrics, Lake Oswego, OR, USA) software. AMPAR EPSC amplitude was measured as the mean amplitude during a 2 ms window around the negative peak. NMDAR EPSC amplitude was recorded in voltage-clamp at +40 mV and measured as the mean positive amplitude between 50–60 ms after onset [28]. fEPSP size was estimated using a linear regression of the 0.8 ms initial slope. The presynaptic volley was not allowed to change more than 15%. mEPSC analyses was based on recordings at a given membrane potential for 4–5 minutes before and after AMPA application and were analyzed using Mini-analysis program (version 5.1.4; Synaptosoft, Decatur, GA, USA).

### Statistical analysis

Data are expressed as means ± s.e.m. Two-tailed t-tests or Kolmogorov-Smirnov tests were used to test for statistical significance.

## Results

### The agonist AMPA causes depression of AMPAR-mediated transmission in the developing rat hippocampus and occludes further stimulus-induced depression

**Figure 1:**
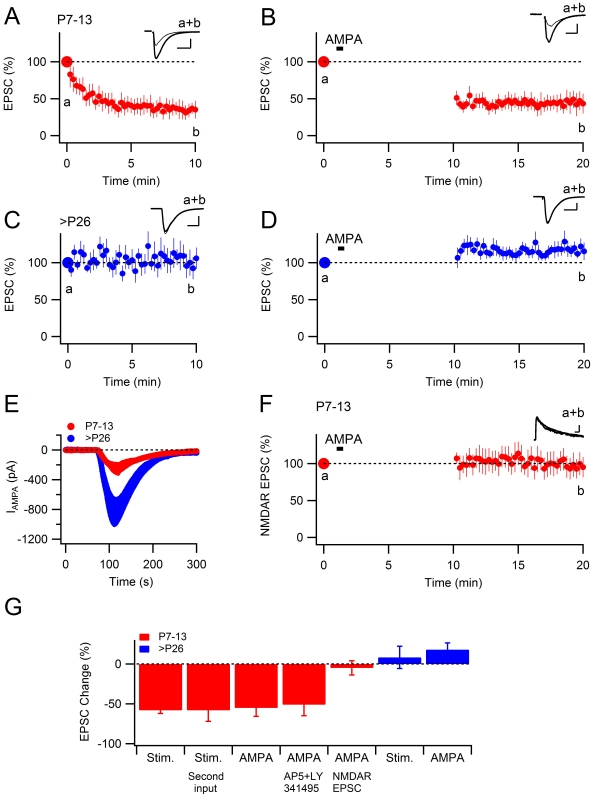
AMPA-induced depression exclusively at developing synapses. **A** and **C**, Whole-cell recordings from CA1 pyramidal cells in acute slices from P7–13 day-old rats (**A**) and >P26 day-old rats (**C**). Synaptic stimulation (0.2 Hz) results in depression of AMPAR-mediated synaptic transmission in developing (**A**, *n* = 16), but not in mature (**C**, *n* = 8) synapses. **B** and **D**, Brief application of AMPA (5 µM, 30 s) results in depression of AMPAR-mediated synaptic transmission in developing (**B**, *n* = 7), but not in mature (**D**, *n* = 6) synapses. Three synaptic stimuli were applied before the application of AMPA and the synaptic stimulation was interrupted for 10 minutes during the agonist application. **E**, Whole-cell currents evoked by bath application of AMPA (5 µM, 30 s) were larger in mature pyramidal cells (P7–13, *n* = 7; >P26, *n* = 8). **F**, Effects of AMPA application on NMDAR-mediated synaptic transmission in developing synapses (*n* = 5). **G**, Summary of stimulus and agonist-induced depression using the whole-cell patch clamp configuration. All data are means ± s.e.m. Scale bars represent 200 pA and 20 ms. Representative EPSCs are shown superimposed above the graphs. Thick traces are averages taken during the first three stimuli. Thin traces are averages during the last minute of recordings, as indicated in the figure.

First, we confirmed [21], [27] that activation of previously unstimulated synapses in acute rat hippocampal slices from young animals (postnatal day (P) 7–13) resulted in synaptic depression (Fig. 1A, G). Using whole-cell recording and voltage-clamp at −70 mV, 120 stimuli at 0.2 Hz resulted in depression to 42±5% of the initial EPSC (*n* = 16, *P*<0.001). To examine whether it is possible to produce this stimulus-induced depression by activating AMPARs directly, we applied (RS)-AMPA hydrobromide (5 µM) to the bath for 30 s after having established the initial synaptic strength with three stimuli (Fig. 1B, G). Application of AMPA caused a transient inward current (250±84 pA, *n* = 7) that returned to baseline within three minutes (Fig. 1E). When synaptic stimulation re-started, the AMPA EPSC was depressed to 45±11% of the initial level (*n* = 7, *P*<0.001). Following the AMPA-induced depression, no further stimulus-induced depression was produced (Fig. 1B), showing that the chemically induced AMPAR depression occludes the stimulus-induced depression.

### At mature synapses, neither agonist application, nor synaptic stimulation induces depression

In contrast, when tested in slices from mature rats (P26–46) there was no stimulus-induced depression (Fig 1C; 108±12% of the initial level, *n* = 8, *P* = 0.80). There was also no AMPA-induced depression at this age (Fig. 1D, G; 118±8% of the initial level, *n* = 6, *P* = 0.08), although the transient inward current was substantially larger in slices from mature rats (Fig. 1E; 835±203 pA, *n* = 6). To test whether AMPA-induced depression at developing synapses was specific to AMPAR-mediated transmission, we repeated the experiment in Fig. 1B, but instead measured the evoked NMDAR EPSC recorded at +40 mV. In accordance with previous results, showing that stimulus-induced synaptic depression at low stimulus frequencies (0.033–0.2 Hz) does not affect synaptic NMDAR signaling [21], [26], [27], there was no depression of evoked NMDAR EPSCs after the AMPA application (Fig 1F, G, ; 95±9% of the initial level, *n* = 5, *P* = 0.68). These results demonstrate that agonist activation of AMPARs induces a depression of AMPAR-mediated transmission exclusively at developing synapses, leaving NMDARs unaffected, and that this AMPA-induced depression occludes subsequent stimulus-induced depression.

### Agonist-induced depression occurs independently of NMDAR or mGluR activation

Stimulus-induced AMPAR silencing at previously unstimulated synapses in the developing hippocampus has been shown to occur independently of NMDAR or mGluR activation [21], [22], [27], [29]. When tested in three experiments, AMPA-induced depression was also not reduced in the combined presence of the NMDAR and mGluR antagonists D-AP5 (50 µM) and LY 341495 (100 µM) (51±14% of the initial level, *n* = 3, *P*<0.01, Fig. 1G). Moreover, as shown below, the AMPA-induced depression could also be induced in the presence of the Na^+^ channel blocker tetrodotoxin (TTX), suggesting that isolated AMPAR activation is sufficient for the induction of AMPA-induced depression. A requirement of AMPAR activation for the induction of the stimulus-induced depression is suggested from experiments showing that this synaptic depression is restricted to the activated synapses [21], [27], [29]. In the present study we confirmed the input specificity by stimulating a second synaptic input to the cell recorded from, after having induced stimulus-induced depression in a first synaptic input. The amount of depression in the second synaptic input was similar to the amount of depression in the first synaptic input (first input to 37±11% of the initial level vs. second input to 42±13%, *n* = 4, *P* = 0.73; Fig. 1G), as would be expected if the stimulus-induced depression was input specific.

### Mutual occlusion between agonist-induced depression and stimulus-induced depression

**Figure 2:**
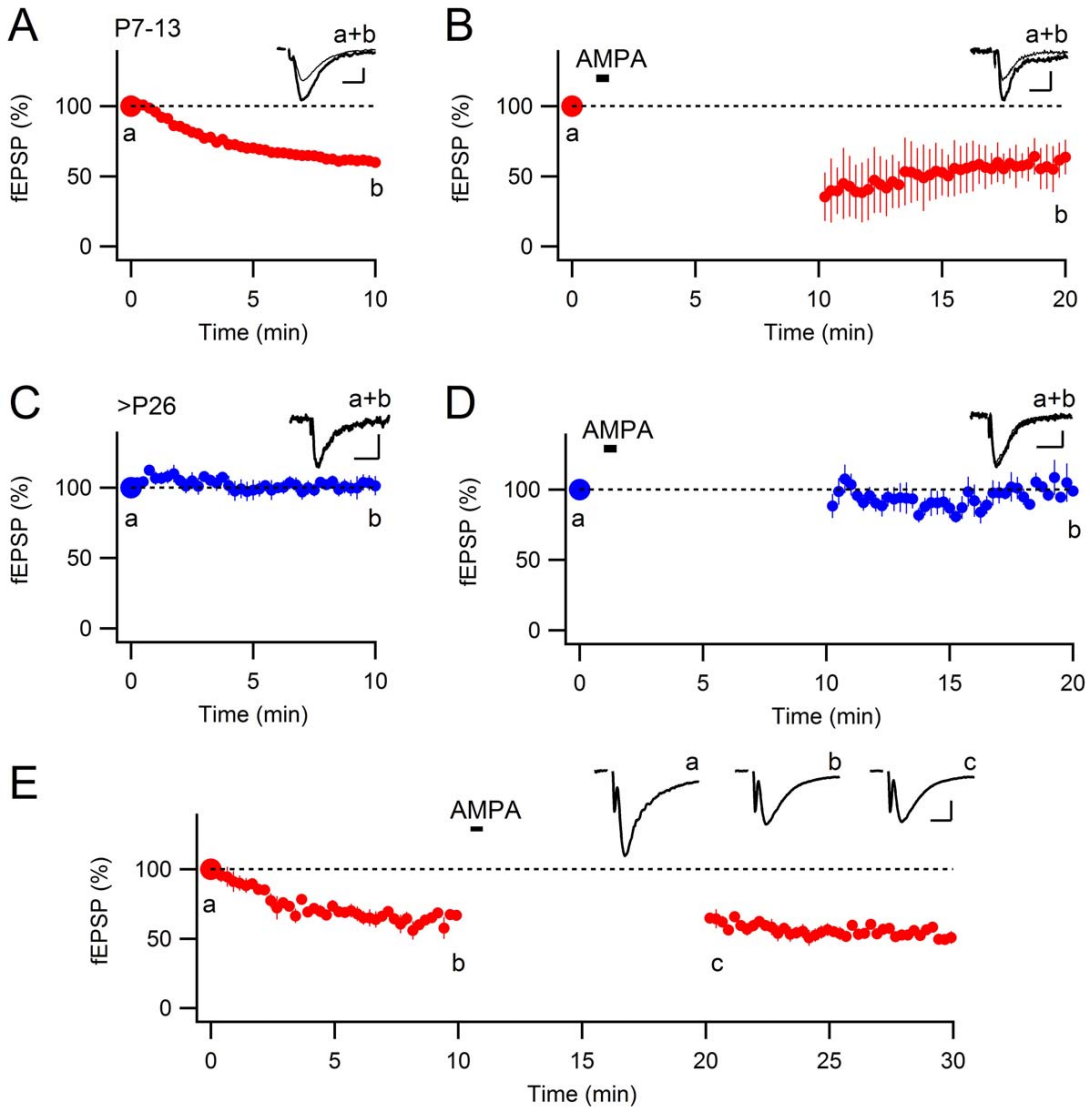
Extracellular field recordings of AMPA-induced depression. **A** and **C**, Extracellular field recordings showing depression of AMPAR-mediated synaptic transmission in developing (**A**, *n* = 17), but not in mature (**C**, *n* = 8) synapses. **B** and **D**, Brief application of AMPA (5 µM, 30 s) results in depression of AMPAR-mediated synaptic transmission in developing (**B**, *n* = 7), but not in mature (**D**, *n* = 6) synapses. Three synaptic stimuli were applied before the application of AMPA and the synaptic stimulation was interrupted for 10 minutes during the agonist application. **E**, Stimulus-induced depression (120 stimuli at 0.2 Hz) is followed by a brief application of AMPA (5 µM, 30 s) in acute slices from P7–13 day-old rats using extracellular field recordings (*n* = 7). All data are means ± s.e.m. Scale bars represent 200 µV and 20 ms. Representative fEPSPs are shown superimposed above the graphs. In **A**–**D**, thick traces are averages taken during the first three stimuli and thin traces are averages during the last minute of recordings, as indicated in the figure. In **E**, fEPSPs from indicated time points are shown.

**Figure 3:**
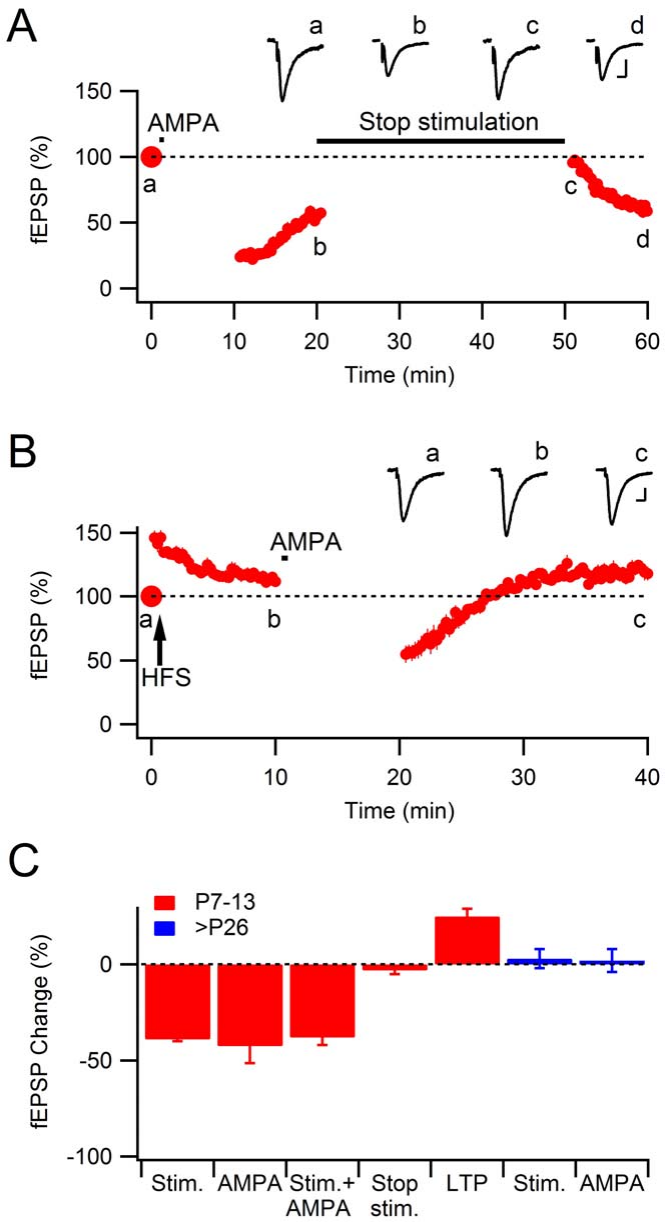
Reversibility and LTP after agonist-induced depression. **A**, Summary data for reversibility experiments in which AMPA-induced depression was followed by a pause of stimulation for 30 minutes (*n* = 6), showing reversal of synaptic signaling to the initial (100%) level. **B**, Summary data for LTP experiments (*n* = 6) in which AMPA was applied 10 min after five trains of high-frequency stimulation (HFS, each consisting of 20 stimuli at 50 Hz, 20 s intertrain interval), showing that further agonist-induced depression was prevented. **C**, Summary of stimulus and agonist-induced depression in extracellular field recordings. All data are means ± s.e.m. Scale bars represent 200 µV and 20 ms. Representative fEPSPs are shown superimposed above the graphs as indicated in the figure.

To exclude that AMPA-induced depression is related to the whole-cell recording conditions we repeated the above experiments using field recordings. In accordance with the results using whole-cell recordings, stimulus-induced depression was present at developing synapses (Fig. 2A, 3C; 61±1% of the initial level, *n* = 17, *P*<0.01), but not at mature synapses (Fig. 2C, 3C; 102±5% of the initial level, *n* = 8, *P* = 0.75). Similarly, AMPA-induced depression was also present at developing synapses (Fig. 2B, 3C; 58±9% of the initial level, *n* = 7, *P*<0.001), but not at mature synapses (Fig. 2D, 3C; 101±6% of the initial level, *n* = 6, *P* = 0.91). These experiments corroborate the results summarized in Fig. 1, demonstrating that AMPA-induced depression occludes stimulus-induced depression.

We also wanted to test whether the reverse is true, i.e. if stimulus-induced depression occludes AMPA-induced depression. Thus, using field recordings, we first induced stimulus-induced depression by 120 stimuli at 0.2 Hz, and then applied AMPA. As shown in Fig. 2E, the stimulus-induced depression (64±3%, *n* = 7) largely occluded any further AMPA-induced depression (62±4, *n* = 7, *P* = 0.133, using paired t-test).

### Reversibility of agonist-induced depression

Previously, it has been shown that stimulus-induced AMPAR is largely reversible after stimulus interruption for 20–30 minutes [27], [30], in other words, this depression needs to be maintained by synaptic activation. Fig. 3A summarizes experiments illustrating that also the AMPA-induced depression is reversible in the absence of synaptic activation. After having induced the AMPA-induced depression, a pause of the stimulation for 30 minutes resulted in a reversal of the depression (to 97±2% of the initial level, *n* = 6, *P* = 0.78; Fig. 3A, C). The resumed synaptic stimulation after the pause resulted in a stimulus-induced depression of comparable magnitude as in naïve control slices (cf. Fig. 2A). This result further strengthens the idea that agonist-induced and stimulus-induced depressions are based on the same mechanisms.

### Developmental LTP prevents agonist-induced AMPAR depression

We have previously shown that stimulus-induced depression is reversed by LTP and, in contrast to the reversal caused by interrupting synaptic activation (cf. Fig. 3A), LTP results in a more stable synaptic transmission [21], [22]. To examine whether LTP also prevents agonist-induced depression we adopted a protocol in which the synapses are exposed to high-frequency stimulation (five 20 impulse trains at 50 Hz, delivered at 20 seconds interval) after only three baseline stimuli (to prevent stimulus-induced depression, but still obtain a baseline value) [22]. Such high-frequency stimulation results in a short-term potentiation that decays within some 10 minutes followed by a more stable “potentiation” that lingers around the initial baseline [22]. In the present experiments we interrupted the stimulation and applied AMPA (5 µM, 30 s) 10 minutes after the high-frequency stimulation, and resumed stimulation 10 minutes later (Fig. 3B). After an initial depression, the fEPSP returned to a level similar to that preceding the application of AMPA (to 123±4% of the initial level, *n* = 6, *P*<0.001; Fig. 3B, C), indicating that the induction of LTP had stabilized these developing synapses to the extent that the agonist-induced depression was prevented.

### Agonist-induced depression in the developing hippocampus is expressed as a total AMPAR silencing in a large number of synapses

**Figure 4:**
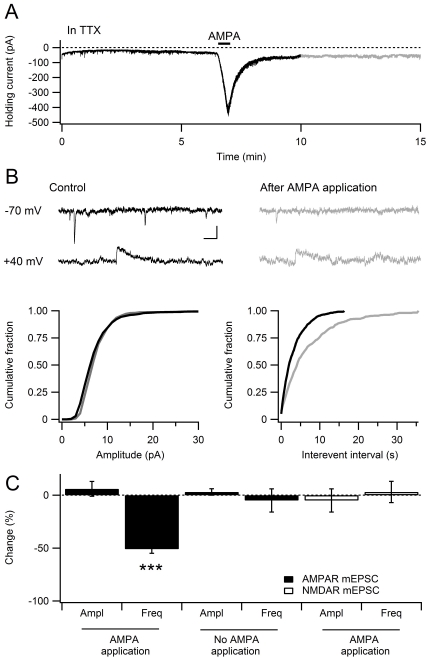
Selective decrease in the frequency of miniature AMPAR-mediated EPSCs after application of AMPA. **A**, Continuous recording of AMPAR mEPSCs in the presence of 500 nM TTX before and after application of AMPA. 5 µM AMPA was applied for 30 s as indicated. **B**, Effects of AMPA application on cumulative distribution of mEPSC amplitude and inter event-interval (*n* = 8). Scale bars represent 10 pA and 10 ms. Representative traces recorded at −70 mv and +40 mV are shown on top. **C**, Bar graph summarizing the effect of AMPA application on AMPAR and NMDAR mEPSCs (*** *P*<0.001). All data are means ± s.e.m.

The stimulus-induced depression is expressed as a total and selective silencing of AMPAR-mediated transmission in a subset of synapses, and with no change in quantal amplitude in the rest of the synapses [21], [26]. To test whether the AMPA-induced depression shares these expression properties, miniature EPSCs (mEPSCs) were recorded in the presence of TTX (500 nM) before and after bath application of AMPA in P7–13 slices (Fig. 4A). The cumulative distribution of mEPSC amplitudes was not different after the application of AMPA (Fig. 4B; *n* = 8; *P*>0.05, Kolmogorov–Smirnov test), but the distribution of inter event-interval was on the other hand significantly altered (Fig. 4B; *n* = 8; *P*<0.05, Kolmogorov–Smirnov test). The frequency of mEPSCs was reduced to 49±3% of control (Fig. 4C; baseline, 0.47±0.09 Hz; after AMPA application, 0.23±0.04 Hz, *n* = 8, *P*<0.001), whereas mEPSC amplitude was not affected (Fig. 4C; baseline, 8.19±0.38 pA; after AMPA application, 8.47±0.40 pA, *n* = 8, *P* = 0.52). To exclude that prolonged whole-cell recordings by itself leads to a lowered mEPSC frequency, 15 minutes recordings were performed without AMPA application, but did not result in any reduction of the mEPSC frequency, as previously reported [31] (Fig. 4C; 103±4%, *n* = 5, *P* = 0.86). These results are in agreement with previous findings indicating that stimulus-induced depression is expressed as a selective decrease in the quantal content reported exclusively by the AMPARs, with no change in the quantal size of non-silenced synapses [21], [26]. To evaluate if the decrease in mEPSC frequency was specific to AMPAR signaling, NMDAR mEPSCs were recorded at +40 mV (Fig. 4B, lower traces) before and after AMPA application. Consistent with the results using evoked NMDA EPSCs (Fig. 1E), there was no change in the frequency or the amplitude of NMDAR mEPSCs after the AMPA application (Fig. 4C; frequency, 95±11% of baseline; amplitude, 103±11% of baseline, *n* = 7, *P* = 0.80 and 0.84). The lack of change in NMDAR mEPSCs and the decrease in frequency of AMPAR mEPSCs shows that a transient exposure to AMPA results in AMPAR silence at around half of the developing synapses.

## Discussion

Our study shows that agonist activation of AMPARs is a sufficient triggering stimuli for inducing synaptic depression at developing hippocampal synapses, whereas mature synapses are resistant to either agonist- or stimulus-induced depression. Prior induction of LTP at the developing synapses prevented the agonist-depression, indicating that LTP contributes to stabilize synaptic transmission at immature glutamate synapses. The agonist-induced depression was associated with a fifty percent reduction in AMPA mEPSC frequency, but no effect on NMDA mEPSCs, indicating that it is expressed as silencing of AMPAR-mediated transmission in about half of the synapses during the second postnatal week. These properties are shared with those reported from the stimulus-induced AMPAR silencing [21], [22], [24], [26], [27], [29]. There was also a mutual occlusion between the AMPA-induced and the stimulus-induced depression. Our results thus suggest that agonist-induced removal of synaptic AMPARs is a basis for activity-dependent synaptic depression at developing glutamate synapses, and that LTP can protect these developing synapses against this agonist-induced removal of synaptic AMPARs.

In contrast to developing synapses, mature synapses did not exhibit agonist- or stimulus-induced synaptic AMPAR silencing. During the second postnatal week, about half of the synapses were AMPAR silenced in response to the agonist, with no change in NMDAR signaling. The remaining synapses were resistant to AMPAR silencing in response to a brief agonist application and thus resembled mature synapses. This population of synapses equipped with more stable basal AMPAR-mediated transmission apparently increased with development, possibly as a result of an accumulation of LTP in the synaptic population. It is noteworthy that we did not observe any reduction in the AMPA mEPSC amplitude in response to AMPA application, indicating synapses either maintain, or lose, all their AMPARs after exposure to the agonist. What constitutes the differences in signaling properties between these two populations of synapses are therefore of particular interest.

Previous studies in neuronal cultures have demonstrated that application of AMPA results in AMPAR removal from the synapse [17], [18], [32]. Moreover, AMPA application causes dissociation between AMPAR and the transmembrane AMPA receptor regulatory proteins (TARPs) [33] and increases lateral diffusion into the perisynaptic domain [34] where AMPARs are internalized into early endosomes [35]. The internalized AMPARs are either sorted to the late endosomal system for later degradation in the lysosome or to recycling endosomes [35]. AMPAR recycling appears necessary for stable synaptic transmission since blockade of this recycling result in an activity-dependent rundown of the AMPA EPSCs [36], [37]. In the framework of this model for constitutive AMPAR recycling [18]–[20] our results suggest that immature glutamate synapses lack rapid constitutive recycling of internalized AMPARs, and therefore lack stable basal synaptic transmission.

LTP unsilences AMPAR silenced synapses and equip these synapses with more stable basal synaptic transmission [21], [22], [38]–[40] (Fig. 3B). It is thus possible that an important feature of developmental LTP is to activate fast endosomal AMPAR recycling at immature synapses. Indeed, chemical induction of LTP has been shown to accelerate AMPAR recycling via the recycling endosome [18] and to promote the mobilization of recycling endosomes into spines [41]. Since about half of the synapses in our study were resistant to agonist-induced silencing of AMPAR transmission it is conceivable that they had already been equipped with mature recycling machinery, possibly through an earlier LTP-induction.

The present results indicate that AMPAR activation is both necessary and sufficient for AMPAR removal at immature synapses. AMPAR silencing induced by AMPAR activation, without concomitant activation of NMDARs, or mGluRs, is however formally no LTD since it is slowly reversible (within 20 minutes) in the absence of synaptic activation [21], [22], [27], [29] (Fig. 3A). These studies however suggested that additional NMDAR and/or mGluR activation results in a more robust synaptic depression possibly by strengthening both the induction and the maintenance of the depression. For the more mature synapses, where AMPAR activation alone is insufficient to cause AMPAR removal, appropriate NMDAR and/or mGluR activation may be the signal that renders the synapse susceptible for agonist-induced AMPAR removal. Indeed, AMPAR activation has been shown to be necessary for the induction of LTD also at more mature synapses [42]. Furthermore, additional NMDAR and/or mGluR activation may stabilize the removal of the AMPARs by providing a signal for sorting the endosome to lysosomal degradation, possibly by changing the phosphorylation status of AMPAR subunits [18]. In this way NMDAR and/or mGluR activation may stabilize the AMPAR silent state of a depressed synapse during development [43], and perhaps drive the synapse one step further towards elimination [14]. In summary, our results suggest that during early brain development, synaptic activity results in agonist-induced AMPAR silencing at a large number of synapses, probably representing an important step in developmental synapse remodeling by constituting an early, reversible signal towards synapse pruning.

## References

[1] Malinow R, Malenka RC (2002). AMPA receptor trafficking and synaptic plasticity.. Annu Rev Neurosci.

[2] Derkach VA, Oh MC, Guire ES, Soderling TR (2007). Regulatory mechanisms of AMPA receptors in synaptic plasticity.. Nat Rev Neurosci.

[3] Yashiro K, Philpot BD (2008). Regulation of NMDA receptor subunit expression and its implications for LTD, LTP, and metaplasticity.. Neuropharmacology.

[4] Yasuda H, Barth AL, Stellwagen D, Malenka RC (2003). A developmental switch in the signaling cascades for LTP induction.. Nat Neurosci.

[5] Groc L, Gustafsson B, Hanse E (2006). AMPA signalling in nascent glutamatergic synapses: there and not there!. Trends Neurosci.

[6] Hall BJ, Ghosh A (2008). Regulation of AMPA receptor recruitment at developing synapses.. Trends Neurosci.

[7] Jensen V, Kaiser KM, Borchardt T, Adelmann G, Rozov A (2003). A juvenile form of postsynaptic hippocampal long-term potentiation in mice deficient for the AMPA receptor subunit GluR-A.. J Physiol.

[8] Hua JY, Smith SJ (2004). Neural activity and the dynamics of central nervous system development.. Nat Neurosci.

[9] Penzes P, Cahill ME, Jones KA, VanLeeuwen JE, Woolfrey KM (2011). Dendritic spine pathology in neuropsychiatric disorders.. Nat Neurosci.

[10] Rakic P, Bourgeois JP, Eckenhoff MF, Zecevic N, Goldman-Rakic PS (1986). Concurrent overproduction of synapses in diverse regions of the primate cerebral cortex.. Science.

[11] Lendvai B, Stern EA, Chen B, Svoboda K (2000). Experience-dependent plasticity of dendritic spines in the developing rat barrel cortex in vivo.. Nature.

[12] Verhage M, Maia AS, Plomp JJ, Brussaard AB, Heeroma JH (2000). Synaptic assembly of the brain in the absence of neurotransmitter secretion.. Science.

[13] Katz LC, Shatz CJ (1996). Synaptic activity and the construction of cortical circuits.. Science.

[14] Hanse E, Taira T, Lauri S, Groc L (2009). Glutamate synapse in developing brain: an integrative perspective beyond the silent state.. Trends Neurosci.

[15] Collingridge GL, Peineau S, Howland JG, Wang YT (2010). Long-term depression in the CNS.. Nat Rev Neurosci.

[16] Lissin DV, Carroll RC, Nicoll RA, Malenka RC, von Zastrow M (1999). Rapid, activation-induced redistribution of ionotropic glutamate receptors in cultured hippocampal neurons.. J Neurosci.

[17] Lin JW, Ju W, Foster K, Lee SH, Ahmadian G (2000). Distinct molecular mechanisms and divergent endocytotic pathways of AMPA receptor internalization.. Nat Neurosci.

[18] Ehlers MD (2000). Reinsertion or degradation of AMPA receptors determined by activity-dependent endocytic sorting.. Neuron.

[19] Passafaro M, Piech V, Sheng M (2001). Subunit-specific temporal and spatial patterns of AMPA receptor exocytosis in hippocampal neurons.. Nat Neurosci.

[20] Shi S, Hayashi Y, Esteban JA, Malinow R (2001). Subunit-specific rules governing AMPA receptor trafficking to synapses in hippocampal pyramidal neurons.. Cell.

[21] Xiao MY, Wasling P, Hanse E, Gustafsson B (2004). Creation of AMPA-silent synapses in the neonatal hippocampus.. Nat Neurosci.

[22] Abrahamsson T, Gustafsson B, Hanse E (2008). AMPA silencing is a prerequisite for developmental long-term potentiation in the hippocampal CA1 region.. J Neurophysiol.

[23] Abrahamsson T, Gustafsson B, Hanse E (2005). Synaptic fatigue at the naive perforant path-dentate granule cell synapse in the rat.. J Physiol.

[24] Meng K, Li YH, Zhang L, Li P, Han TZ (2010). Ca2+-permeable AMPA receptors mediate induction of test pulse depression of naive synapses in rat visual cortical slices at early postnatal stage.. Neuroscience.

[25] Kerchner GA, Nicoll RA (2008). Silent synapses and the emergence of a postsynaptic mechanism for LTP.. Nat Rev Neurosci.

[26] Riebe I, Gustafsson B, Hanse E (2009). Silent synapses onto interneurons in the rat CA1 stratum radiatum.. Eur J Neurosci.

[27] Abrahamsson T, Gustafsson B, Hanse E (2007). Reversible synaptic depression in developing rat CA3 CA1 synapses explained by a novel cycle of AMPA silencing-unsilencing.. J Neurophysiol.

[28] Groc L, Gustafsson B, Hanse E (2002). Spontaneous unitary synaptic activity in CA1 pyramidal neurons during early postnatal development: constant contribution of AMPA and NMDA receptors.. J Neurosci.

[29] Strandberg J, Wasling P, Gustafsson B (2009). Modulation of low-frequency-induced synaptic depression in the developing CA3-CA1 hippocampal synapses by NMDA and metabotropic glutamate receptor activation.. J Neurophysiol.

[30] Strandberg J, Gustafsson B (2011). Critical and complex role of N-methyl-D-aspartate receptors in long-term depression at CA3-CA1 synapses in the developing hippocampus.. Neuroscience.

[31] Hsia AY, Malenka RC, Nicoll RA (1998). Development of excitatory circuitry in the hippocampus.. J Neurophysiol.

[32] Carroll RC, Beattie EC, Xia H, Luscher C, Altschuler Y (1999). Dynamin-dependent endocytosis of ionotropic glutamate receptors.. Proc Natl Acad Sci U S A.

[33] Morimoto-Tomita M, Zhang W, Straub C, Cho CH, Kim KS (2009). Autoinactivation of neuronal AMPA receptors via glutamate-regulated TARP interaction.. Neuron.

[34] Tardin C, Cognet L, Bats C, Lounis B, Choquet D (2003). Direct imaging of lateral movements of AMPA receptors inside synapses.. EMBO J.

[35] Kennedy MJ, Ehlers MD (2006). Organelles and trafficking machinery for postsynaptic plasticity.. Annu Rev Neurosci.

[36] Luscher C, Xia H, Beattie EC, Carroll RC, von Zastrow M (1999). Role of AMPA receptor cycling in synaptic transmission and plasticity.. Neuron.

[37] Nishimune A, Isaac JT, Molnar E, Noel J, Nash SR (1998). NSF binding to GluR2 regulates synaptic transmission.. Neuron.

[38] Liao D, Hessler NA, Malinow R (1995). Activation of postsynaptically silent synapses during pairing-induced LTP in CA1 region of hippocampal slice.. Nature.

[39] Isaac JT, Nicoll RA, Malenka RC (1995). Evidence for silent synapses: implications for the expression of LTP.. Neuron.

[40] Durand GM, Kovalchuk Y, Konnerth A (1996). Long-term potentiation and functional synapse induction in developing hippocampus.. Nature.

[41] Park M, Salgado JM, Ostroff L, Helton TD, Robinson CG (2006). Plasticity-induced growth of dendritic spines by exocytic trafficking from recycling endosomes.. Neuron.

[42] Scholz R, Berberich S, Rathgeber L, Kolleker A, Kohr G (2010). AMPA receptor signaling through BRAG2 and Arf6 critical for long-term synaptic depression.. Neuron.

[43] Montgomery JM, Madison DV (2002). State-dependent heterogeneity in synaptic depression between pyramidal cell pairs.. Neuron.

